# Real-world anticoagulatory treatment after percutaneous mitral valve repair using MitraClip: a retrospective, observational study on 1300 patients

**DOI:** 10.1007/s00392-022-01988-2

**Published:** 2022-02-26

**Authors:** Christopher Hohmann, Marion Ludwig, Jochen Walker, Christos Iliadis, Jan-Hendrik Schipper, Stephan Baldus, Roman Pfister

**Affiliations:** 1 Faculty of Medicine, Department III for Internal Medicine, Heart Center, University Hospital of Cologne, Kerpener Str. 62, 50937 Cologne, Germany; 2 InGef - Institute for Applied Health Research Berlin GmbH, Berlin, Germany

**Keywords:** MitraClip, Anticoagulation, NOAC, Antithrombotic therapy, Real-world

## Abstract

**Aims:**

This study sought to investigate current anticoagulatory treatment patterns and clinical outcome in patients undergoing transcatheter mitral valve repair (MitraClip).

**Methods and results:**

In a retrospective study of a German claims database (InGef research database), anticoagulatory treatment regimens were assessed using any drug prescription post discharge within the first 90 days after MitraClip procedure. Clinical events between 30 days and 6 months were examined by treatment regime. The study population comprised 1342 patients undergoing MitraClip procedure between 2014 and 2018. 22.4% received antiplatelet monotherapy, 20.8% oral anticoagulation (OAC) plus antiplatelet therapy, 19.4% OAC monotherapy, 11.7% dual antiplatelet therapy, 2.8% triple therapy and 21.0% did not receive any anticoagulatory drugs. 63% of patients with OAC received non-vitamin-K antagonist oral anticoagulants (NOAC). A total of 168 patients were newly prescribed OAC after MitraClip, of whom 12 patients (7.1%) had no diagnosis of atrial fibrillation or venous thromboembolism. 40% of patients with OAC prior to MitraClip did not have any OAC after MitraClip. The adjusted risk of all-cause mortality was significantly increased in patients with no anticoagulatory treatment (HR 3.84, 95% CI 2.33–6.33, *p* < 0.0001) when compared to antiplatelet monotherapy whereas the other regimes were not significantly different.

**Conclusions:**

This large real-world data analysis demonstrates a heterogeneous spectrum of prescriptions for anticoagulant therapies after MitraClip. Considering relevant differences in clinical outcome across treatment groups, major effort is warranted for controlled trials in order to establish evidence-based recommendations on anticoagulatory treatment after percutaneous mitral valve repair.

**Graphical abstract:**

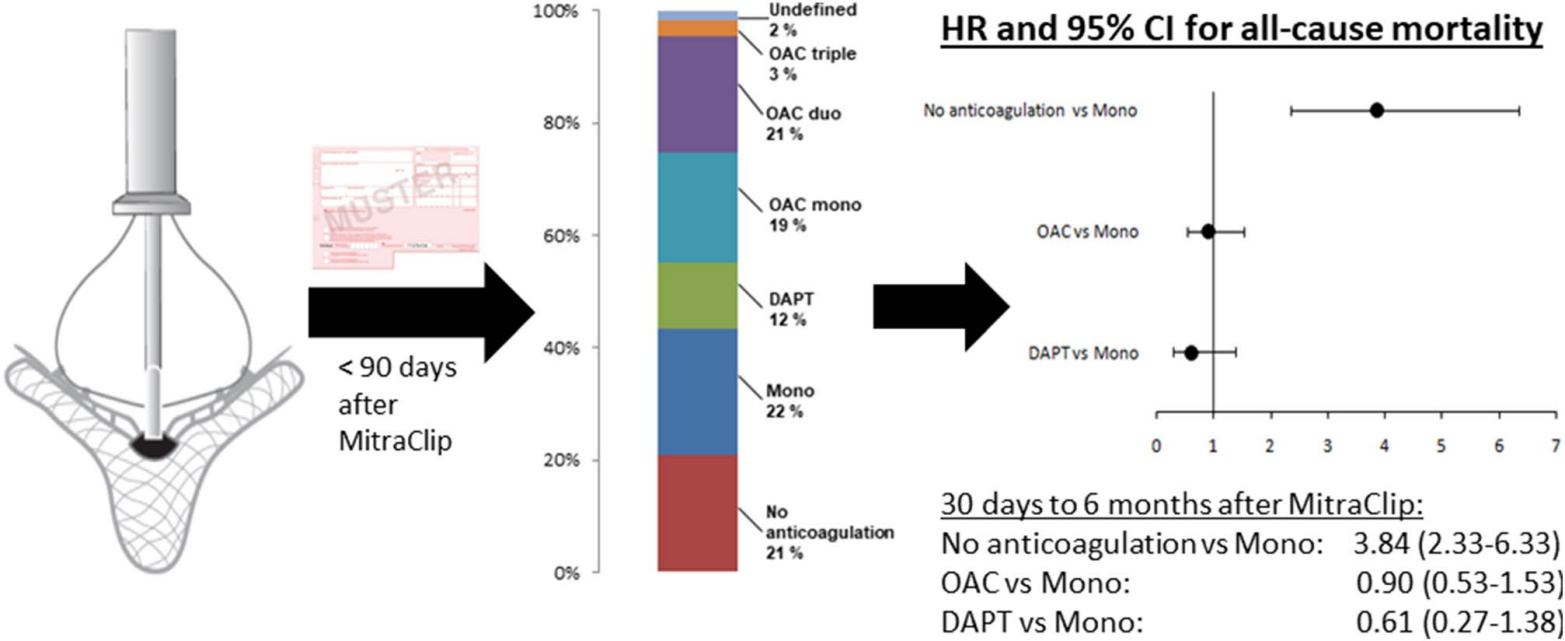

**Supplementary Information:**

The online version contains supplementary material available at 10.1007/s00392-022-01988-2.

## Introduction

Percutaneous mitral valve repair using MitraClip is an established technique for the treatment of severe mitral regurgitation in patients considered to be high risk for surgical repair or replacement [1, 2]. Although MitraClip implantation is a generally safe procedure, there remains the clinical challenge of balancing risks for thromboembolic events with bleeding complications particularly during the early postprocedural period.

In this regard, patients with severe mitral regurgitation are likely to have atrial fibrillation (AF) with a presence of up to 63% and an indication for permanent oral anticoagulation [3]. Furthermore, the implanted MitraClip poses an increased thromboembolic risk until the endothelialization of the device is completed. Some reported cases of device thrombosis and atrial thrombosis immediately post-MitraClip placement indicate a particular thrombogenic risk in the early postprocedural period [4, 5]. Therefore, extrapolated from experiences with septal occluder devices a regimen of aspirin at a dose of 325 mg daily for 6 months to 1 years combined with clopidogrel at a dose of 75 mg daily for 1 month was administered in trials of patients undergoing MitraClip procedure without indication for oral anticoagulation [6–8]. On the other hand, patients undergoing MitraClip procedure are often frail and show several comorbidities contributing to bleeding complications [9, 10]. In this respect, a high incidence of bleeding events immediately post-intervention of up to 22% were described [11]. As a consequence, physicians may be reluctant to perform intensive oral anticoagulation in patients after MitraClip implantation in clinical routine.

To date, there are no evidence-based recommendations for the anticoagulant therapy after MitraClip implantation. In patients with sinus rhythm, the administration of dual antiplatelet therapy using aspirin and clopidogrel for up to six months has become best practice standard [8, 12]. In the presence of risk factors such as atrial fibrillation, oral anticoagulation should be continued after the procedure [13, 14], but there is uncertainty on whether additional antiplatelet therapy is necessary during the first months. Current ESC/EACTS and ACC/AHA guidelines give a Class IIa indication for temporary oral anticoagulation within the first months after surgical mitral valve repair even in patients with no additional risk factors [1, 15]. However, it is unclear whether interventional reconstruction using MitraClip is associated with a comparable risk to surgical reconstruction.

For decades, vitamin-K antagonists (VKA) were the standard of choice for long-term prevention of thrombosis after valve repair or replacement. In recent years, non-vitamin-K antagonist oral anticoagulants (NOAC) have been shown to be superior in comparison to VKA in patients with nonvalvular atrial fibrillation [16, 17]. However, in the context of mitral valve repair including MitraClip and atrial fibrillation data on NOACs are lacking.

Taken together, there is substantial uncertainty regarding the anticoagulatory therapy in the individual patient after percutaneous mitral valve repair given the lack of prospective trials. Considering the major impact of antiplatelet and oral anticoagulation therapy on bleeding risk in elderly and multimorbid patients, the aim of the present study was to assess current real-world treatment patterns in patients undergoing MitraClip therapy. For this purpose, we used longitudinal German Statuary Health Insurance claims data to evaluate drug prescriptions and short-term clinical effectiveness and safety data.

## Methods

### Study design and data source

This non-interventional retrospective cohort study was based on data from the InGef—Institute for Applied Health Research database. The InGef research database is based on anonymized, health claims data of approximately 7 million insurees, comprehending about 10% of the statutory health insured population in Germany. It provides longitudinal information on the utilization of services on a case-by-case individual level. In brief, the database includes demographic information, information on outpatient healthcare services and data related to hospital treatment, including admission and discharge dates, diagnoses, operations and interventions (OPS codes) as well as prescription and dispensation of reimbursed medications. All diagnoses in the database were coded according to the International Classification of Diseases (ICD), Tenth Revision, German Modification (ICD10-GM). Data on outpatient prescriptions of reimbursed drugs comprise information on the prescription, the date of prescription and the pharmaceutical reference number. The database has a high external validity regarding morbidity, mortality, and drug prescriptions [18].

All patient identifiers were either fully encrypted or removed from the database which is therefore compliant with the German data protection regulations. As no patient contact was made and patient information was deidentified, Institutional Review Board Approval was not required.

### Study population

We identified adult patients (≥ 18 years of age) with available information on age and gender who received MitraClip for the first time within the study period from 01 January 2014 to 31 December 2018 using OPS code 5-35a.41. The date of MitraClip was defined as the index date. Patients were required to have a continuous health plan enrollment for 6 months pre-index (baseline period used for assessment of baseline characteristics of morbidity and medication pre-MitraClip) as well as 6 months post-index or death. Patients with a history of mechanical heart valve replacement or previous MitraClip and patients with NOAC dosages that are not approved for prophylaxis of thromboembolic events in Germany were excluded.

Depending on prescriptions of any of the following antithrombotic or anticoagulation drugs at any time within the period of 90 days post-MitraClip (postprocedural anticoagulation regime), patients were grouped into a total of 6 mutually exclusive regimens: no antithrombotic/anticoagulatory treatment, single antiplatelet therapy (aspirin, clopidogrel, prasugrel, or ticagrelor), dual antiplatelet therapy (aspirin in combination with either clopidogrel, prasugrel or ticagrelor), single oral anticoagulation (with a NOAC [apixaban, edoxaban, dabigatran, or rivaroxaban] or VKA [phenprocoumon]) (OAC mono), oral anticoagulation (with a NOAC [apixaban, edoxaban, dabigatran, or rivaroxaban] or VKA [phenprocoumon]) plus aspirin or clopidogrel (OAC duo) and oral anticoagulation (with a NOAC [apixaban, edoxaban, dabigatran, or rivaroxaban] or VKA [phenprocoumon]) plus aspirin and clopidogrel (OAC triple). Patients who received a prescription that could not be categorized into one of the aforementioned regimens were classified as undefined therapy, for instance patients who were prescribed both phenprocoumon and a NOAC or more than one distinct NOAC.

### Clinical endpoints

The effectiveness outcomes were (1) major adverse cardiovascular events (MACE) defined as a combined endpoint of cardiovascular mortality, myocardial infarction and ischemic stroke, and (2) death from any cause. The safety outcomes were intracranial bleeding, major extracranial bleeding and gastrointestinal bleeding. Intracranial bleeding was defined as subarachnoidal bleeding, intracerebral bleeding and other non-traumatic and traumatic intracranial bleeding. Major extracranial bleeding was defined as a bleeding with anaemia, hemothorax, conjunctival hemorrhage, retinal hemorrhage, unspecified, recurrent and persistent hematuria, hemorrhage from respiratory passages, hemarthrosis as well as other abnormal uterine and vaginal bleeding. The outcomes were identified using ICD-10-GM codes in the hospital main and secondary discharge diagnosis as well as confirmed ambulatory diagnoses (Supplementary Table 1). The prementioned endpoints were evaluated for the period of > 30 days up to 6 months after MitraClip, respectively, since events during the early postprocedural period are usually attributable to the procedure itself and less likely due to the anticoagulation medication which is usually initiated several days after the procedure.

### Statistical analysis

Data analysis was performed by InGef. The primary outcome was the postprocedural anticoagulation regime. Baseline characteristics of the study population were reported as percentages or mean ± standard deviation. Statistical significance across groups was examined using chi-square test for categorical variables and one-way analysis of variance (ANOVA) for metric variables, respectively. Secondary outcomes were agreement with best practice standards with respect to anticoagulation treatment after MitraClip procedure (i.e. DAPT or any OAC regime), and postprocedural termination of OAC and postprocedural initiation of OAC without justifying diagnosis. Secondary analysis on the prevalence of postprocedural anticoagulation regimes was performed excluding patients who died within the first 30 days after MitraClip. The reason was the lack of data on anticoagulation treatment during the hospitalization and potential drug intake provided by the hospital for the early post-discharge days and in consequence the uncertainty of assignment of such patients to respective anticoagulation regimes, in particular the “no anticoagulation” category.

Unadjusted event rates were calculated by dividing the number of events by the person time and were reported per 100 person-years. Cox proportional-hazard regression models were used to estimate treatment effects of dual antiplatelet therapy, any OAC (total of OAC mono, OAC duo, OAC triple) and no anticoagulation for all-cause mortality in the period > 30 days up to 6 months after MitraClip using single antiplatelet therapy as the reference group. Models were adjusted for prespecified baseline demographics and clinical factors only for all-cause mortality which had a sufficient number of events. Variables for inclusion in the model were selected based on established evidence on the effect of the specific variable on the choice of treatment and mortality. To estimate the magnitude of underdetection of postprocedural prescription of anticoagulation using a 90 days interval, we examined how many patients received a follow-up prescription of VKA/NOAC or antiplatelet agent, respectively, within an observation period of 120 days after their last preprocedural prescription in the two patient groups with intake of single antiplatelet therapy or VKA/NOAC prior to MitraClip and no respective follow-up prescription within the 90 days post MitraClip. All statistical analyses were performed using SAS software version 9.4 (SAS Institute GmbH, Heidelberg, Germany).

## Results

### Postprocedural anticoagulatory treatment

The study population comprised 1,342 patients with MitraClip between 2014 and 2018. Mean age was 76 ± 9 years and 63% were male. Mean Charlson Comorbidity Index was 5.1 + 3.0. The most frequent comorbidities were arterial hypertension (91%), congestive heart failure (78%), coronary heart disease (73%), atrial fibrillation (62%), and renal insufficiency (51%) (Table 1).

**Table 1:** Baseline characteristics of patients after MitraClip

	Monotherapy (ASS/Clopidogrel)	DAPT	OAC mono	OAC duo	OAC triple	No anticoagulation	Undefined therapy	Total study population	Overall p-value
	*n* = 301	*n* = 157	*n* = 261	*n* = 279	*n* = 37	*n* = 282	*n* = 25	*n* = 1,342	
Age (mean ± SD)	75.7 (8.9)	74.6 (10.4)	77.8 (7.4)	77.0 (8.2)	77.9 (6.8)	75.9 (9.7)	78.2 (6.9)	76.4 (8.8)	0.00
Male (%)	199 (66.1)	103 (65.6)	152 (58.2)	168 (60.2)	25 (67.6)	179 (63.5)	15 (60.0)	841 (62.7)	0.48
Charlson Comorbidity Index (mean ± SD)	5.4 (3.0)	5.0 (3.0)	4.9 (3.1)	4.9 (2.8)	4.8 (3.5)	5.1 (2.9)	5.1 (3.1)	5.1 (3.0)	0.33
CHA_2_DS_2_-VASc-Score (mean ± SD)	4.5 (1.4)	4.3 (1.5)	4.7 (1.3)	4.7 (1.5)	4.4 (1.5)	4.5 (1.5)	5.0 (1.4)	4.6 (1.4)	0.05
modified HAS-BLED-Score (mean ± SD)	3.1 (1.1)	3.1 (1.2)	3.1 (1.0)	3.0 (1.0)	3.0 (1.0)	3.1 (1.1)	3.1 (1.0)	3.1 (1.1)	0.63
Renal insufficiency (%)	148 (49.2)	84 (53.5)	123 (47.1)	137 (49.1)	23 (62.2)	152 (53.9)	14 (56.0)	681 (50.7)	0.45
Dementia (%)	11 (3.6)	10 (6.4)	15 (5.7)	10 (3.6)	6 (16.2)	13 (4.6)	< 5	67 (5.0)	0.02
History of ischemic stroke/TIA (%)	17 (5.6)	12 (7.6)	17 (6.5)	24 (8.6)	< 5	17 (6.0)	< 5	89 (6.6)	0.32
Myocardial infarction < 12 months (%)	47 (15.6)	20 (12.7)	26 (10.0)	28 (10.0)	< 5	36 (12.8)	< 5	163 (12.1)	0.02
Coronary heart disease (%)	234 (77.7)	105 (66.9)	192 (73.6)	198 (71.0)	27 (73.0)	203 (72.0)	19 (76.0)	978 (72.9)	0.30
History of coronary angioplasty (PCI)/Stenting (%)	6 (2.0)	5 (3.2)	5 (1.9)	< 5	< 5	< 5	< 5	24 (1.8)	0.04
Congestive heart failure (%)	236 (78.4)	111 (70.7)	208 (79.7)	215 (77.1)	25 (67.6)	230 (81.6)	21 (84.0)	1046 (78.0)	0.11
Hypertension (%)	276 (91.7)	134 (85.4)	243 (93.1)	253 (90.7)	33 (89.2)	256 (90.8)	23 (92.0)	1218 (90.8)	0.26
Cancer (%)	80 (26.6)	29 (18.5)	60 (23.0)	58 (20.8)	5 (13.5)	73 (25.9)	5 (20.0)	310 (23.1)	0.23
Arteriosclerosis (%)	68 (22.6)	34 (21.7)	48 (18.4)	61 (21.9)	10 (27.0)	64 (22.7)	8 (32.0)	293 (21.8)	0.66
Diabetes mellitus (%)	139 (46.2)	74 (47.1)	117 (44.8)	128 (45.9)	16 (43.2)	116 (41.1)	14 (56.0)	604 (45.0)	0.74
Obesity (%)	68 (22.6)	40 (25.5)	69 (26.4)	68 (24.4)	8 (21.6)	60 (21.3)	6 (24.0)	319 (23.8)	0.85
History of any bleeding event (%)	54 (17.9)	23 (14.6)	51 (19.5)	39 (14.0)	6 (16.2)	62 (22.0)	6 (24.0)	241 (18.0)	0.21
Moderate or severe hepatic insufficiency (%)	< 5	< 5	< 5	< 5	< 5	< 5	< 5	5 (0.4)	0.54
Atrial fibrillation (%)	146 (48.5)	35 (22.3)	206 (78.9)	207 (74.2)	23 (62.2)	202 (71.6)	19 (76.0)	838 (62.4)	0.00
Previous venous thromboembolism (%)	13 (4.3)	< 5	13 (5.0)	13 (4.7)	< 5	11 (3.9)	< 5	53 (3.9)	0.12
ACE inhibitors/angiotensin receptor antagonist (%)	130 (43.2)	78 (49.7)	123 (47.1)	115 (41.2)	17 (46.0)	136 (48.2)	13 (52.0)	612 (45.6)	0.50
NSAIDs (%)	54 (17.9)	27 (17.2)	49 (18.8)	46 (16.5)	5 (13.5)	46 (16.3)	< 5	229 (17.1)	0.38
Beta-blocker (%)	248 (82.4)	110 (70.1)	225 (86.2)	236 (84.6)	27 (73.0)	223 (79.1)	22 (88.0)	1091 (81.3)	0.00
Diuretics (%)	257 (85.4)	125 (79.6)	227 (87.0)	238 (85.3)	28 (75.7)	248 (87.9)	22 (88.0)	1145 (85.3)	0.17
Antipsychotics (%)	10 (3.3)	8 (5.1)	14 (5.4)	9 (3.2)	5 (13.5)	9 (3.2)	< 5	56 (4.2)	0.06
Proton pump inhibitors (%)	158 (52.5)	84 (53.5)	135 (51.7)	155 (55.6)	23 (62.2)	145 (51.4)	14 (56.0)	714 (53.2)	0.86
Statins (%)	172 (57.1)	74 (47.1)	139 (53.3)	148 (53.0)	16 (43.2)	139 (49.3)	16 (64.0)	704 (52.5)	0.22
All- cause mortality < 30 days after procedure (event rate)	< 5 (n.a.)	< 5 (n.a.)	< 5 (n.a.)	< 5 (n.a.)	< 5 (n.a.)	50 (50.3)			

Baseline characteristics were determined in the 180 days prior to the respective MitraClip procedure. Data are *n* (%), unless otherwise indicated. Data are not shown in cells that contain fewer than five patients. *ACE* angiotensin-converting enzyme, *ASS* acetylsalicylic acid, *NSAID* non-steroidal anti-inflammatory drug, *SD* standard deviation

**Fig. 1:**
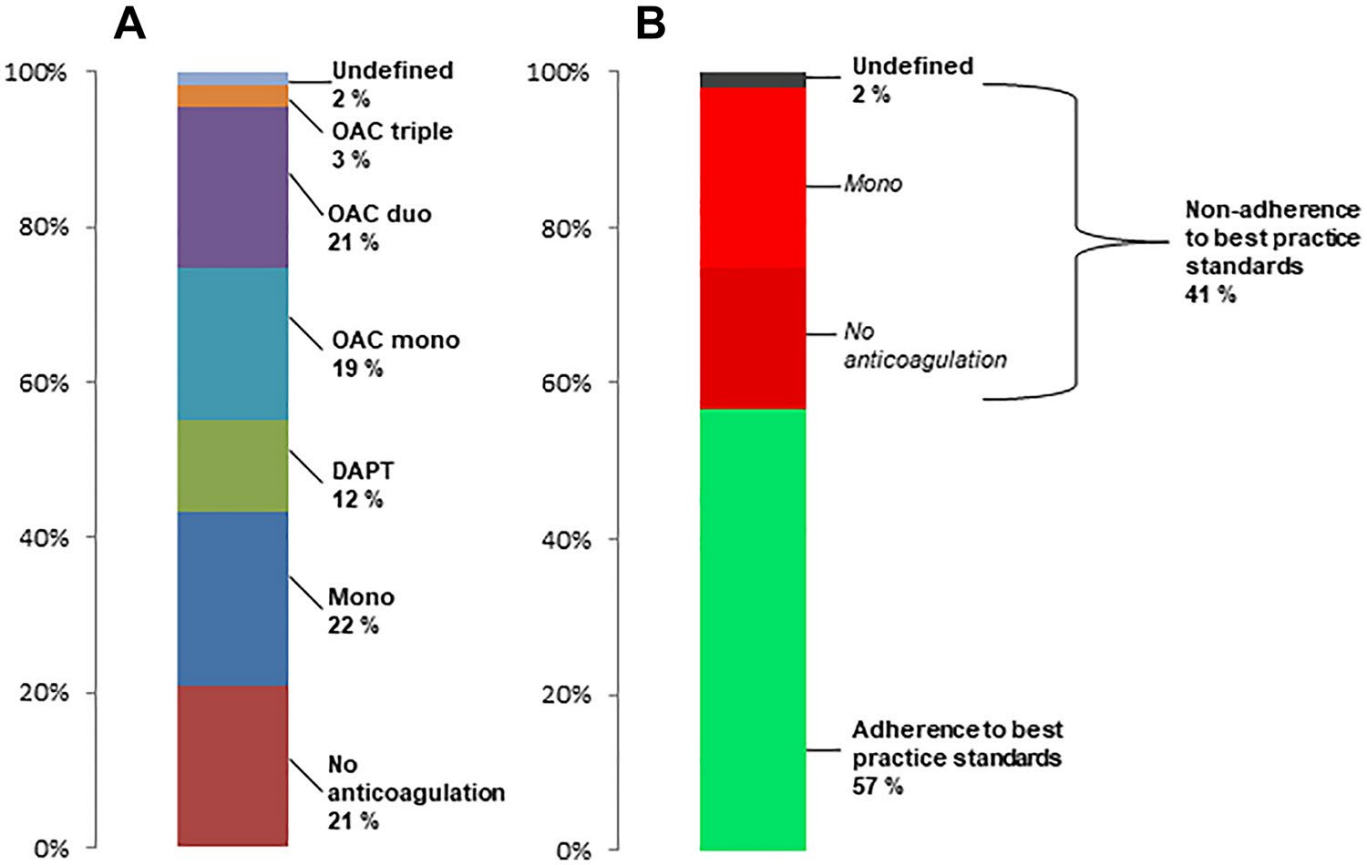
Frequency of post-MitraClip anticoagulatory treatment regimes in the total population (**A**) and rate of treatments not in agreement with current best practice standards in patients surviving first 30 days after MitraClip (**B**)

Based on all drug prescriptions during the first 90 days after MitraClip the postprocedural anticoagulation regime was assessed for every patient (Fig. 1A). The majority of patients received antiplatelet monotherapy (*n* = 301, 22.4%), followed by OAC duo (*n* = 279, 20.8%), OAC mono (*n* = 261, 19.4%), DAPT (*n* = 157, 11.7%) and OAC triple (*n* = 37, 2.8%). A total of 282 patients (21.0%) did not receive any prescription of an anticoagulatory drug and 25 patients (1.9%) had an undefined anticoagulation regime based on the combination of drug prescriptions.

Baseline characteristics of the total study population and by postprocedural anticoagulation regime are presented in Table 1. The frequency of individual characteristics differed significantly between treatment groups with regard to age, dementia, myocardial infarction < 12 months ago, history of coronary angioplasty (PCI)/stenting, atrial fibrillation and intake of beta-blocker. For example, atrial fibrillation was 3- to 4-fold more common in patients with a regime including OAC. Regarding platelet inhibition, single and dual antiplatelet therapy was associated with a more frequent history of coronary angioplasty and myocardial infarction < 12 months.

### Agreement with best practice standards of postprocedural anticoagulatory therapy

43.4% of patients had either no anticoagulation or single antiplatelet therapy. When excluding patients who died during the first 30 days after MitraClip (*n* = 54), still 23.3% had only antiplatelet monotherapy and 18.0% had no prescription for anticoagulatory drugs (Fig. 1B). Furthermore, 28% of all patients who survived the first 30 days had NOAC after MitraClip, corresponding to 63% of patients with postprocedural OAC.

**Fig. 2:**
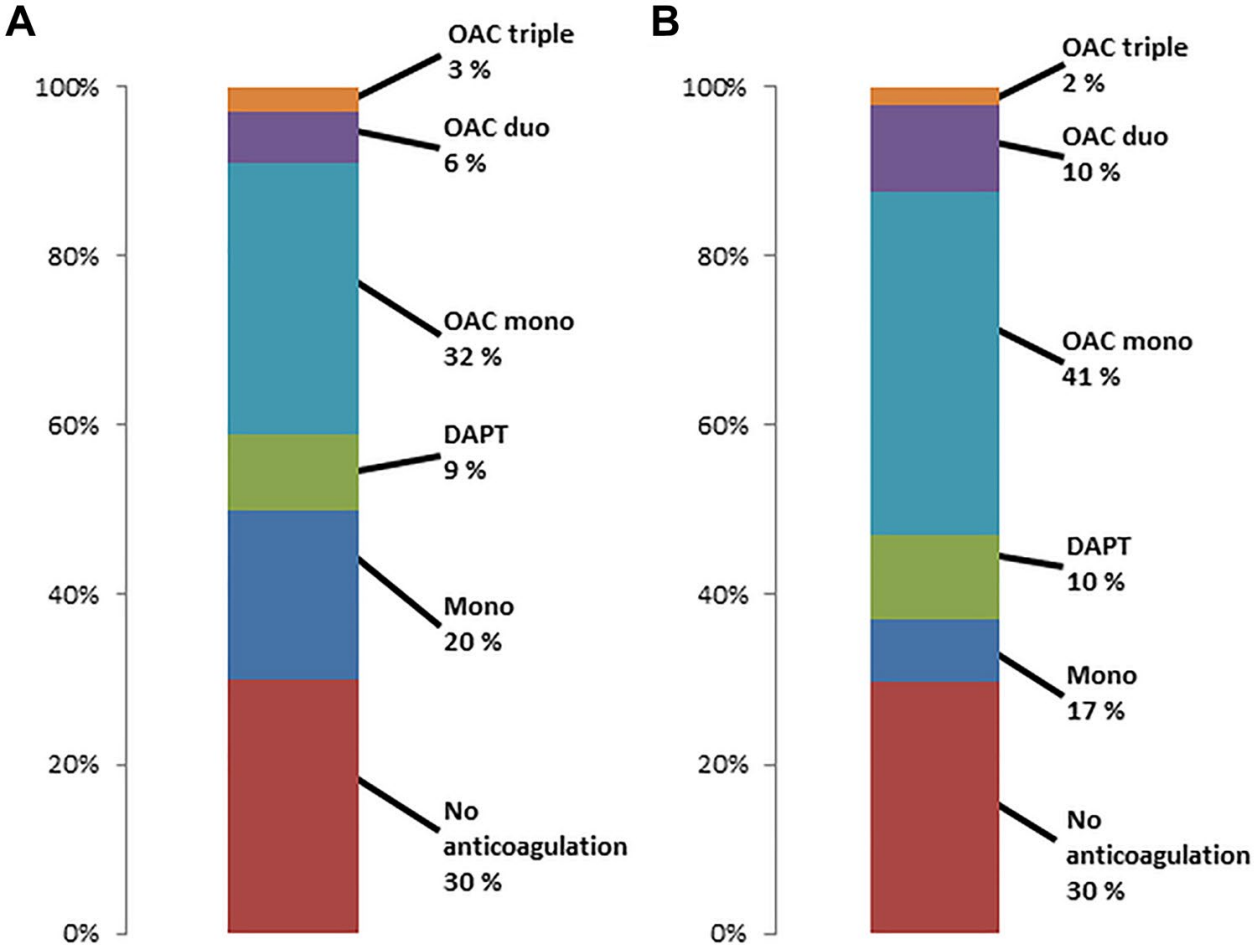
Preprocedural anticoagulatory regimes in patients surviving first 30 days after MitraClip with (**A**) postprocedural antiplatelet monotherapy and (**B**) postprocedural no antiplatelet or anticoagulation therapy

Figure 2 shows the distribution of preprocedural anticoagulation regimes for patients who survived the first 30 days after MitraClip and had postprocedural antiplatelet monotherapy (A) or no anticoagulation (B). A total of 30% of patients with postprocedural antiplatelet monotherapy had no anticoagulatory medication prior to MitraClip, and 50% had a more extensive anticoagulation such as DAPT or OAC. 30% of patients without any postprocedural anticoagulation had no anticoagulatory medication prior to MitraClip, and about 53% had OAC prior to MitraClip.

### Changes in OAC and type of OAC from pre- to postprocedural

**Fig. 3:**
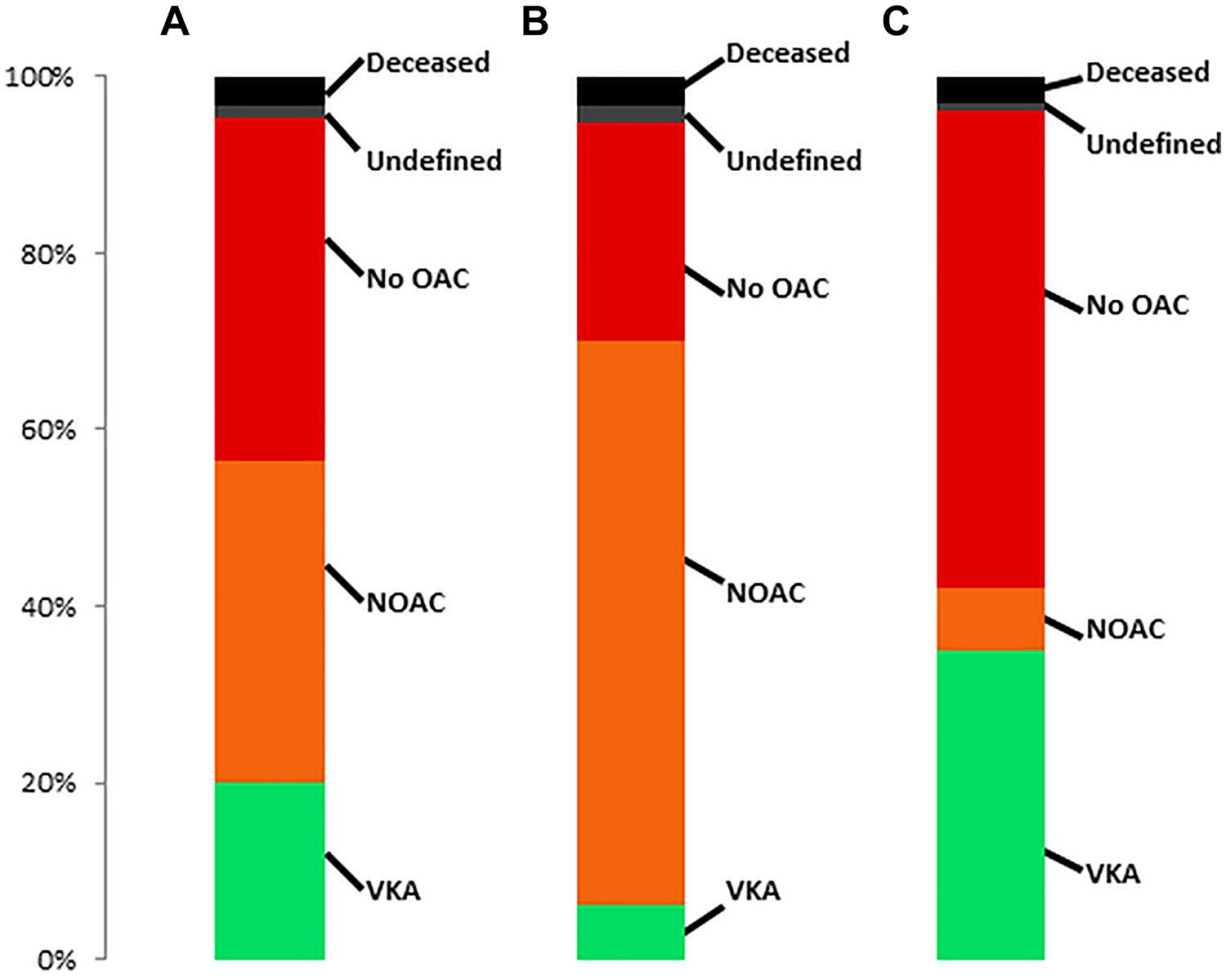
Frequency of post-MitraClip anticoagulatory treatment regimes in patients with (**A**) OAC prior to MitraClip, (**B**) NOAC prior to MitraClip and (**C**) VKA prior to MitraClip

685 (51.0%) patients had any OAC before MitraClip, with similar use of NOAC (*n* = 356, 52.0%) and VKA (*n* = 329, 48.0%). The respective postprocedural type of OAC in patients with any preprocedural OAC, preprocedural NOAC and preprocedural VKA is presented in Fig. 3. Almost 40% of patients did not have any OAC prescription post-procedurally. The rate was significantly higher in patients with prior VKA treatment compared to prior NOAC treatment (*p* < 0.001). When excluding patients who died during the first 30 days after MitraClip, still 39% did not have any postprocedural OAC prescription.

A total of 168 patients were newly prescribed OAC after MitraClip, of whom 89 patients (53%) were treated with a NOAC and 79 patients (47%) with VKA, respectively. 12 patients (7.1%) out of these received OAC for the first time without a diagnosis of atrial fibrillation or venous thromboembolism.

### Effectiveness and safety outcomes

Table 2 displays the number of effectiveness and safety outcome events occurring between 30 and 180 days after the procedure and the respective unadjusted event rates per 100 person-years according to postprocedural anticoagulation regime. MACE were generally rare, with slightly higher event rates in the combined treatment group with OAC mono/OAC duo. The highest event rates of death from all cause were observed in patients with no anticoagulation, followed by a comparable rate in patients with antiplatelet therapy and treatment groups containing oral anticoagulation (OAC mono, OAC duo). After adjusting for baseline confounders (age, gender, renal insufficiency, history of PCI/Stenting, atrial fibrillation, history of any bleeding event, history of myocardial infarction < 12 months, congestive heart failure, beta-blocker, dementia), in comparison to single antiplatelet therapy the risk of all-cause mortality was not significantly different in patients with DAPT (HR 0.61, 95% CI 0.27–1.38, *p* = 0.23) and OAC containing regimes (HR 0.90, 95% CI 0.53–1.53, *p* = 0.70). However, no anticoagulation was associated with a significantly increased risk of all-cause mortality (HR 3.84, 95% CI 2.33–6.33, *p* < 0.001).

**Table 2:** Event numbers and rates (per 100 person-years) of efficacy and safety endpoints according to post-MitraClip anticoagulant treatment

	> 30 days up to 6 months after procedure					
	Monotherapy/DAPT		OAC mono/OAC duo		No anticoagulation	
	*N* events	Event rate	N events	Event rate	N events	Event rate
MACE	< 5	n.a	8	3.0	< 5	n.a
All-cause mortality	31	14.2	37	14.4	57	57.4
Combined bleeding endpoint (intracranial/extracranial/gastrointestinal bleeding)	16	7.3	8	3.1	8	5.9
Gastrointestinal bleeding	12	5.4	6	2.3	< 5	n.a

Data are not shown in cells that contain fewer than five patients

Abbreviations: *DAPT* dual antiplatelet therapy, *MACE* major adverse cardiac event, *OAC* oral anticoagulation, *SE* systemic embolism, *n.a.* not available

The bleeding events were mainly driven by gastrointestinal bleedings. Event rates were higher in patients with antiplatelet monotherapy/DAPT than in patients with OAC mono/OAC duo. In patients with no anticoagulation, event rates for bleeding were low and comparable with those treated with single antiplatelet inhibition.

### Sensitivity analysis

When using a time period of 120 days after the last preprocedural prescription to detect a postprocedural drug prescription for patients with OAC prior to MitraClip and no OAC prescription within the 90 days interval, a total of 9 patients (6.1%) were ultimately issued a follow-up prescription for OAC and 139 patients (93.9%) still remained without prescription. For 24 patients who received single antiplatelet therapy before MitraClip and no antithrombotic prescription within the 90 days postprocedural interval, 1 (4.2%) patient was prescribed aspirin or clopidogrel during the modified observation period.

## Discussion

The present study is the first to report anticoagulatory treatment regimens after MitraClip in a large real-world cohort. More than one-third of the patients received an OAC containing regime with a NOAC portion of 63%, 22% of patients received antiplatelet monotherapy, 12% DAPT and 20% had no antiplatelet or anticoagulation drug prescription. About 40% of patients with OAC prior to MitraClip had no OAC prescription within the first 90 days after procedure. Twelve (7.1%) out of 168 patients with newly prescribed OAC after MitraClip had no justifying diagnosis of atrial fibrillation or venous thromboembolism. The risk of all-cause mortality was significantly increased in patients without any antiplatelet and anticoagulation therapy after MitraClip.

So far data on clinical reality of anticoagulatory treatment after MitraClip are scarce. In the COAPT trial, 48% of patients received oral anticoagulation with a NOAC proportion of 16%. Regarding platelet inhibition, the prescription of aspirin and P2Y12 inhibitors was 67% and 36%, respectively, at 30 days after implantation [12]. Currently, the use of antiplatelets and anticoagulants for patients receiving the MitraClip device is only recommended on an empirical basis. In our real-world cohort, about 22% of patients had antiplatelet monotherapy. On average these patients had a clinical low risk profile for bleeding and 30% of these patients did not have any anticoagulation prior to MitraClip. This might suggest that the underlying reason for antiplatelet monotherapy is at least in part uncertainty due to the lack of trial evidence and also a lack of clear expert recommendations rather than concern for bleeding risk.

The use of NOACs in patients with indication for OAC is compelling also in the post MitraClip scenario due to the ease of use and the superior safety profile reported in randomized controlled trials for patients with nonvalvular atrial fibrillation [19, 20]. Our findings support this assumption in showing that 63% of patients with OAC after MitraClip use NOACs. However, evidence supporting the efficacy of NOAC in patients after percutaneous mitral valve repair is lacking. This is not trivial because in previous trials a remarkable portion of patients of up to 25% showed an increased transmitral pressure gradient indicating mild to moderate mitral valve stenosis after MitraClip implantation [21–23]. In the context of atrial fibrillation, a valvular etiology must be acknowledged for which NOACs are not approved, and therefore this circumstance requires special attention when prescribing oral anticoagulation after MitraClip implantation. In a prospective observational registry Seeger et al. demonstrated a significantly lower combined endpoint of all-cause mortality, all stroke, and rehospitalization for congestive heart failure or myocardial infarction at 30 days after MitraClip implantation for patients treated with apixaban plus aspirin compared with antiplatelet therapy only. However, this study aimed to compare apixaban with antiplatelet therapy in order to prevent early thromboembolic events in patients with sinus rhythm and an apixaban dose not approved for atrial fibrillation. Taken together, these findings strongly highlight the need for controlled trials on NOAC in patients with atrial fibrillation after percutaneous mitral valve repair [24].

Almost 40% of patients with OAC prior to MitraClip had no OAC prescription after MitraClip, with higher rates in VKA than in NOAC treated patients. This termination rate is more than twofold higher when compared for instance to data reported for patients with atrial fibrillation undergoing PCI [25]. A potential explanation is more severe access site complications in MitraClip compared to PCI, albeit we do not have data available on this. Nonetheless, access site complication should not preclude interruption of OAC for up to 90 days and further study is warranted to elucidate underlying causes of OAC termination after MitraClip.

Clear recommendations on additional antiplatelet drugs are lacking for patients with an indication for OAC after MitraClip. In the COAPT trial protocol an additional use of clopidogrel was recommended. In our cohort, only 6% of patients with OAC were treated with a triple therapy including dual antiplatelet therapy in addition to OAC which is in line with the paradigm shift away from triple therapy in the post PCI setting because of excessive bleeding risk [26–29].

A total of 282 patients (21%) were not prescribed any antiplatelet or anticoagulant therapy after MitraClip. When excluding patients with early death, still 18% of the total population had no postprocedural anticoagulatory drug prescription. These patients were characterized by increased morbidity and higher rates of bleeding events in the past compared with other regimes. Of note, however, 68% had prior antiplatelet or OAC therapy which means that the previous anticoagulatory therapy was actively terminated. The aforementioned comorbidity factors are associated with worse outcome after MitraClip [30] which might be one potential explanation for termination of anticoagulatory medication. To what extent the termination of anticoagulatory therapy itself contributed to the increased risk of mid-term mortality cannot be concluded from our data. However, even after accounting for differences in comorbidity, patients with no postprocedural anticoagulatory treatment had the highest mortality across groups. Since postprocedural MACE in these patients were not higher than in the other treatment groups, an overall increased fragility must be assumed in this patient clientele contributing to cardiovascular and non-cardiovascular mortality risk.

### Strength and limitations

The strength of this study is the large and representative sample size reflecting 10% of the German statutory health insured population, and the data completeness with respect to follow-up and drug prescriptions. However, some limitations are inherent to the particular type of data source. Accuracy of patient characteristics depends on quality of coding. Since our conclusions are not dependent on exact absolute frequencies of comorbidities and coding errors may be similar across exposure groups, moderate inaccuracies in coding will not meaningfully influence conclusions. Clinical details of the postprocedural in-hospital course after MitraClip can impact decisions on anticoagulatory treatment and might not be accurately reflected in coded diagnosis, for instance regarding access site status or minor bleeding events. Hence, our findings are mainly descriptive rather than exploratory. Furthermore, the exact start of the postprocedural anticoagulatory treatment regime cannot be exactly assessed with the available data and might differ from the prescription date. Hence, we pragmatically excluded outcome events within the first 30 days after MitraClip because the temporal relation to the treatment regime is unclear. By using prescription claims data for the defined treatment regimens it is not possible to detect termination of drugs before 90 days since most prescriptions provide drug supply for 90 days. Additionally, a switch of individual drugs or regimes within this early time can also not be accurately detected. Overall, this will lead to “undefined regimes” or an overestimation of total anticoagulatory drug intake. In consequence, the substantial undertreatment with respect to clinical standards observed in our cohort would be even more pronounced. In contrast, there was no evidence for relevant underestimation of treatments in our sensitivity analysis.

## Conclusions

This large real-world data analysis demonstrates a heterogeneous spectrum of prescriptions for anticoagulant therapies after MitraClip. Randomized trials are needed in order to establish evidence-based recommendations on anticoagulatory treatment after percutaneous mitral valve repair and to provide data on open questions such as efficacy of NOAC use and necessity of a second antiplatelet drug in patients with OAC. However, the high rate of patients without any anticoagulatory therapy after MitraClip including 40% with prior OAC is of major clinical concern and needs further study since patients do have a considerable mortality risk.

## Supplementary Information

Below is the link to the electronic supplementary material.

Supplementary file4 (DOCX 13 KB)

## Data Availability

The data used in this study cannot be made available in the manuscript, the supplemental files, or in a public repository due to German data protection laws (Bundesdatenschutzgesetz). To facilitate the replication of results, anonymized data used for this study are stored on a secure drive at the InGef—Institute for Applied Health Research Berlin. Access to the raw data used in this study can only be provided to external parties under the conditions of a cooperation contract and can be accessed upon request, after written approval (info@ingef.de), if required.
